# Influence of Physical Attractiveness and Gender on Patient Preferences in Digital Doctor Consultations: Experimental Study

**DOI:** 10.2196/46551

**Published:** 2024-05-30

**Authors:** Xia Wei, Shubin Yu, Changxu (Victor) Li

**Affiliations:** 1 College of Management Shenzhen University Shenzhen China; 2 Department of Communication and Culture BI Norwegian Business School Oslo Norway; 3 Department of Marketing KU Leuven Leuven Belgium

**Keywords:** digital doctor consultations, health care providers, gender stereotype, physical attractiveness, qualification information, experimental, telemedicine, digital consultation, disease severity, sex, gender, gender stereotypes, digital health

## Abstract

**Background:**

The rise of digital health services, particularly digital doctor consultations, has created a new paradigm in health care choice. While patients traditionally rely on digital reviews or referrals to select health care providers, the digital context often lacks such information, leading to reliance on visual cues such as profile pictures. Previous research has explored the impact of physical attractiveness in general service settings but is scant in the context of digital health care.

**Objective:**

This study aims to fill the research gap by investigating how a health care provider’s physical attractiveness influences patient preferences in a digital consultation setting. We also examine the moderating effects of disease severity and the availability of information on health care providers’ qualifications. The study uses signal theory and the sexual attribution bias framework to understand these dynamics.

**Methods:**

Three experimental studies were conducted to examine the influence of health care providers’ physical attractiveness and gender on patient preferences in digital consultations. Study 1 (n=282) used a 2×2 between-subjects factorial design, manipulating doctor attractiveness and gender. Study 2 (n=158) focused on women doctors and manipulated disease severity and participant gender. Study 3 (n=150) replicated study 2 but added information about the providers’ abilities.

**Results:**

This research found that patients tend to choose attractive doctors of the opposite gender but are less likely to choose attractive doctors of the same gender. In addition, our studies revealed that such an effect is more prominent when the disease severity is high. Furthermore, the influence of gender stereotypes is mitigated in both the high and low disease severity conditions when service providers’ qualification information is present.

**Conclusions:**

This research contributes to the literature on medical information systems research and sheds light on what information should be displayed on digital doctor consultation platforms. To counteract stereotype-based attractiveness biases, health care platforms should consider providing comprehensive qualification information alongside profile pictures.

## Introduction

Digital health services have become increasingly important in health care, particularly since the COVID-19 pandemic necessitated the development of new digital consultation systems [1-4]. Previous research indicates that patients often use digital reviews to select health care providers [5]. However, there is limited understanding of how decisions are made when such reviews are not available. In traditional health care settings (ie, offline health care), patients typically choose or are assigned a general practitioner based on their geographical location. In contrast, digital health care settings remove these geographical restrictions, presenting patients with a vast array of choices and different information. This abundance of choices and inconsistent information can make it challenging for patients to compare and select health care providers [6]. For instance, the availability of digital information can lead patients to select doctors based on inherent biases, which can arise from their personal preferences or the way the information is presented on the platform. Given these limitations, patients may resort to evaluating health care providers based on available photographs. This situation raises questions about the influence of a health care provider’s appearance on patient choices and whether gender differences affect these decisions. Previous research indicates that attractive service providers can positively influence consumer attitudes and purchase intentions [7]. High physical attractiveness is often associated with greater credibility and professional ability, affecting consumer attitudes accordingly [8-10]. However, high attractiveness can also have negative effects, such as difficulty in forming same-gender friendships due to competition or eliciting envy and perceived threats among members of the same gender [11-14].

Much of the existing research focuses on general services such as fitness clubs and educational consulting [15]. These findings may not be directly transferable to specialized digital services, such as digital doctor consultations. Such services are categorized as credence services, which are difficult for patients to evaluate due to their complex and specialized nature [16,17]. The challenge of evaluation is further exacerbated in a digital setting where information about the provider’s abilities may be limited.

There is a research gap in understanding how physical appearance affects patient preferences in digital health care settings. This study aims to fill this gap by examining preferences for doctors of varying levels of physical attractiveness in digital consultations. We also consider the moderating effects of disease severity and the availability of information on health care providers’ qualifications to offer both theoretical and practical insights.

Research on doctor selection uses signal theory to explore how patients navigate information asymmetry [18,19]. Text-mining research identified key service features, such as overall service experience and personality traits, that affects patients’ trust and, consequently, consultation volumes [20]. Digital doctor consultation platforms typically display limited information about doctors, such as their names, profile pictures, and titles. The appearance of the doctor may influence patients’ preferences. Prior studies have shown that the attire of physicians influenced patients’ perceptions [21-23]. For example, physicians in white coats were viewed as more experienced and professional than those in casual jackets [24]. Despite this, there is still scant research on how such visual and textual information affect patients’ decision-making.

In non–health care service settings, the impact of physical attractiveness on performance evaluation has shown mixed results. While some studies indicate that higher levels of attractiveness positively influence performance evaluations [25,26], others suggest a negative effect [27]. These mixed outcomes may be attributed to various contextual factors such as gender [28] and service quality conditions [29]. In digital settings, service providers’ physical appearance has been shown to influence customer choice, preference, and purchase intent [30]. For example, in the sharing economy, such as Airbnb, facial characteristics contribute to reputation mechanisms [31]. Studies have found that the perceived trustworthiness and attractiveness of a host’s profile photograph significantly affect Airbnb prices [32,33]. Physical attractiveness also influences digital consumer shopping, with more attractive avatars correlating with higher sales when product involvement is moderate [34].

Previous research indicates that gender differences significantly influence how patients perceive doctors [24,35,36]. The sexual attribution bias (SAB) offers an explanatory framework for these gender effects [37]. SAB leads individuals to attribute the success of same-gender individuals with high attractiveness to luck rather than ability, whereas for opposite-gender individuals, high attractiveness is attributed to ability [38]. This bias manifests in 2 ways: demeaning attractive individuals of the same gender and praising attractive individuals of the opposite gender. Studies have shown that negative vigilance against attractive same-gender individuals is strong and automatic due to intragender competition [39-42].

Unlike general services where attractiveness universally enhances provider popularity [43,44], we posit that in digital doctor platforms, gender plays a significant role in shaping preferences for providers with varying levels of attractiveness. Influenced by same-gender competition, consumers may perceive same-gender providers with high attractiveness as less qualified. Conversely, influenced by mating motivation, consumers may prefer highly attractive providers of the opposite gender. Given the importance of competence in selecting credence service providers [45], SAB suggests that individuals may make derogatory attributions about the competence of same-gender providers with high physical attractiveness.

- *Hypothesis 1*: In digital doctor consultations, people are more likely to perceive a more attractive doctor of the opposite (vs same) gender as more (less) competent, thereby influencing their likelihood of selecting that doctor.

We anticipate that disease severity will modulate the effects of gender and attractiveness on provider selection. Previous research has established a relationship between disease severity and behavior in various health care contexts [46-49]. The Elaboration Likelihood Model posits 2 routes of information processing: central and peripheral, determined by the individual’s level of involvement [50]. In low-involvement situations, attitudes are influenced by simple cues, whereas high involvement leads to a deeper consideration of complex information. Applying this model and stereotype theory, we suggest that in high-involvement scenarios, consumers will scrutinize providers’ abilities more closely, potentially leading to greater influence of SAB on their choices. For example, women may question the competence of attractive women providers, suspecting that their success is due to their appearance rather than merit [51]. This aligns with research showing that physical attractiveness elicits more jealousy among women than men [14]. Conversely, in low-involvement scenarios, the impact of gender on preferences for providers’ physical attractiveness is expected to be less pronounced.

- *Hypothesis 2*: The effect of gender on individual preferences for the doctor’s physical attractiveness is moderated by disease severity. Such an effect is more prominent when disease severity is high (vs low).

Furthermore, a foundational assumption for the effects discussed earlier is that consumers lack additional information about the service providers’ abilities. This is often the case in credence services, where consumers typically have less expertise and access to information compared with noncredence services, making them more reliant on extra information supplied by the provider [52]. Research has shown that the provision of such information can mitigate consumer-perceived risk or uncertainty [53].

- *Hypothesis 3*: The effect of gender on individual preferences for the service provider’s physical attractiveness disappears when information about the doctor’s abilities is present.

## Methods

### Ethical Considerations

This study has received ethical approval (20180420) from the College of Management, Shenzhen University. Informed consent was obtained from all participants. The consent form provided detailed information about the research’s purpose, the involved institutions, the nature of their participation, and the use of their data. Participants were informed of their right to withdraw at any time and the procedures for data removal. The consent process was designed to comply with data protection legislation, ensuring that participants were aware of their rights and the protections in place. Privacy and confidentiality were protected by anonymizing study data. The data were stored securely and processed. For study 1, we paid CN ¥3 (a currency exchange rate of CN ¥7.21=US $1 is applicable.) for each response we collected. For study 2 and study 3, participants received a gift of approximately CN ¥5 for their participation.

### Study 1

#### Overview

Study 1 used a 2 (attractiveness: high vs moderate)×2 (doctor gender: man vs woman) between-subjects factorial experiment. We created a digital doctor consultation scenario where participants were asked to imagine that they had abdominal pain while traveling. As most clinics are closed at night, they decided to consult a digital doctor via a platform called “*Doctor Online*.”

#### Stimuli

**Figure 1:**
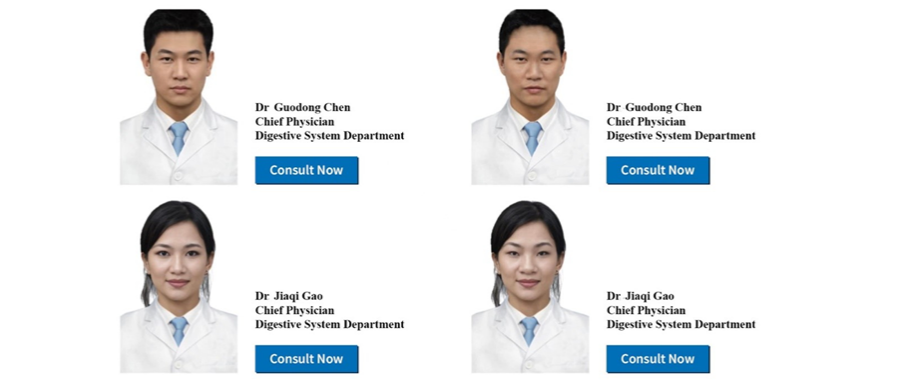
Stimulus used in study 1. These profile pictures were generated by artificial intelligence.

An interface was designed for the platform. In the interface, the doctor’s picture was shown on the left, and their name, title, and department were displayed on the right. There was also a button for digital consultation. To manipulate the physical attractiveness of the doctor, we used an artificial intelligence face generator to generate different faces for male and female doctors (Figure 1). We slightly adapted the physical features of the faces to make them less or more attractive.

#### Pretest

We conducted a pretest to check whether the manipulation was successful. We recruited 111 participants (mean_age_ 30.0, SD 7.86 years; women: n=70, 63.1%) to test the male doctor and 105 participants (mean_age_ 30.5, SD 9.61 years; women: n=74, 70.5%) to test the female doctor via a digital panel service WJX. The results showed that for the male doctor, participants perceived the face in the high attractiveness condition to be more attractive than that in the moderate attractiveness condition (mean_high_ 5.53, SD 0.98 vs mean_moderate_ 4.32, SD 1.09; *F*_1,109_=38.3; *P*<.001; *ƞ*^2^=0.26). For the female doctor, the face in the high attractiveness condition was perceived as more attractive than that in the moderate attractiveness condition (mean_high_ 5.98, SD 0.85 vs mean_moderate_ 4.94, SD 0.95; *F*_1,103_=34.7; *P*<.001; *ƞ*^2^=0.25).

#### Subjects

We recruited 282 respondents via a digital panel service WJX in China. The participants were not necessarily actual patients in this experiment. The mean age of the respondents was 27.8 (SD 9.76) years. In all, 156 (55.3%) of the respondents were women. Based on the respondents’ gender, we created a new variable “gender match.” If the gender of the participant is the same as that of the doctor, we assigned the value 1 (same gender). Otherwise, we coded the variable as 0 (opposite gender). In the end, there were 147 participants in the same gender condition, and 135 participants in the opposite gender condition. The majority of respondents had a bachelor’s degree (n=104, 36.9%), followed closely by those with a graduate degree or higher (n=89, 31.6%). Less than a quarter (n=61, 21.6%) of the respondents had an education level of high school or below. The majority of respondents were students (n=132, 46.8%), followed by those working in industrial or manufacturing enterprises (n=42, 14.9%). Less than 5% (n=17) were either retired or unemployed. The data revealed that a slight majority of respondents (n=153, 54.3%) had experience with digital consultations, while 45.7% (n=129) had not engaged in such services. Tables 1 and 2 show detailed information on educational level and occupational types.

**Table 1:** Distribution of respondents by educational level (study 1; n=282).

Educational level	Frequency, n (%)
Junior high or below	17 (6)
High school or vocational	44 (15.6)
Associate’s degree	28 (9.9)
Bachelor’s degree	104 (36.9)
Graduate or above	89 (31.6)

**Table 2:** Occupational types of respondents (study 1; n=282).

Occupation type	Frequency, n (%)
Commercial or service industry	35 (12.4)
Industrial or manufacturing	42 (14.9)
Self-employed	31 (11)
Retired	8 (2.8)
Unemployed	9 (3.2)
Students	132 (46.8)
Other	25 (8.9)

#### Manipulation Checks

For the manipulation check, we asked the participants to rate the physical attractiveness of the doctor using a single item: *What do you think of the doctor’s physical appearance?* (1—not attractive at all; 7—very attractive). A single item was used to measure the doctor’s perceived competence: *Do you think that this doctor is competent in this job*? (1—no; 7—yes). To assess the participants’ intention to select the doctor, we asked the participants how likely he or she would be to select this doctor for a digital consultation (1—very unlikely; 7—very likely). To rule out alternative explanations, we also measured a number of the doctor’s attributes: perceived seniority (*What do you think about the doctor’s work experience?* 1—very limited; 7—very rich), anticipated embarrassment (*How likely are you to feel embarrassed about consulting this doctor?* 1—not embarrassed at all; 7—very embarrassed), perceived friendliness (*How friendly is this doctor?* 1—not friendly at all; 7—very friendly), and perceived willingness to help (*Do you think this doctor is willing to meet patients’ needs?* 1—not at all; 7—very much). Finally, respondents were asked to provide some relevant demographic information about their age, gender, educational background, and occupation.

### Study 2

#### Overview

In study 2, to reduce the complexity of the experimental design, we focused on women doctors. A between-subjects experiment was designed with 2 independent variables, each with 2 levels. The manipulated factors were disease severity (2 levels: high vs low) and gender of the participant (2 levels: man vs woman).

#### Stimuli

In the experiment, respondents were randomly assigned to 1 of 2 groups of scenarios with different degrees of involvement. They were first asked to read material about a scenario of abdominal pain during travel. The low-involvement group was described as having “slight abdominal pain, the same old symptom, not too worried, decided to consult a doctor.” The high-involvement group heard it described as “severe abdominal pain, lumps when pressing with hands, never encountered such symptoms before, and felt both worried and afraid, decided to consult a doctor immediately.”

**Figure 2:**
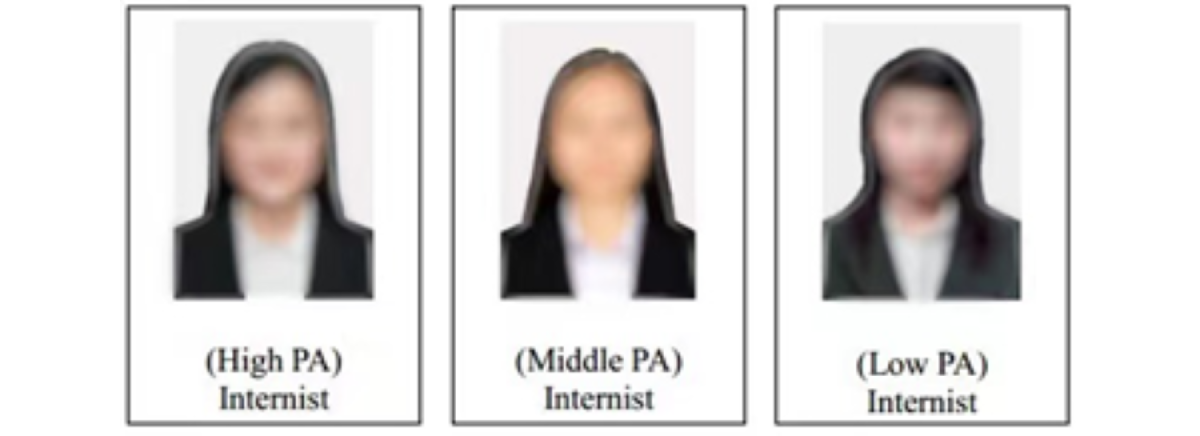
Stimulus used in study 2. The pictures were blurred because of privacy issues. PA: physical attractiveness.

To manipulate physical attractiveness in the dependent variable, we referred to the studies by Heilman and Stopeck [54] and Försterling et al [37], as well as previous studies on the use of photographs as stimuli of attractiveness in job-seeking scenarios [55]. In total, 30 respondents were invited to participate in rating different women service providers’ physical attractiveness. Respondents were asked to use a 5-point Likert scale to rate the attractiveness of providers in ten 2.54-cm photographs. All photographs were selected from several medical websites and their photograph styles were unified. Finally, 3 photographs, representing the mean value, higher than 1 SD and lower than 1 SD, were selected as photographs to represent the 3 different levels of physical attractiveness, as shown in Figure 2.

#### Pretest

We also conducted a second pretest with 28 respondents to check whether confounding factors such as perceived friendliness, perceived patience, perceived seniority, perceived willingness to help, and anticipated embarrassment were controlled among 3 service providers. The results suggested that all 3 service providers were perceived as equally friendly (mean_high PA_ 2.94, SD 1.692; mean_middle PA_ 2.94, SD 2.016; mean_low PA_ 3.75, SD 2.053; *F*_2,25_=0.586; *P*=.56; *ƞ*^2^=0.031), patient (mean_high PA_ 3.19, SD 1.682; mean_middle PA_ 2.88, SD 1.857; mean_low PA_ 3.88, SD 2.475; *F*_2,25_=0.720; *P*=.50; *ƞ*^2^=0.037), experienced (mean_high PA_ 2.44, SD 1.711; mean_middle PA_ 2.81, SD 1.905; mean_low PA_ 3.50, SD 2.390; *F*_2,25_=0.806; *P*=.46; *ƞ*^2^=0.042), willing to help (mean_high PA_ 3.44, SD 1.931; mean_middle PA_ 2.56, SD 1.750; mean_low PA_ 3.50, SD 2.268; *F*_2,25_=1.037; *P*=.37; *ƞ*^2^=0.053), and embarrassing to consult with (mean_high PA_ 3.38, SD 1.996; mean_middle PA_ 3.44, SD 2.007; mean_low PA_ 3.25, SD 1.488; *F*_2,25_=0.025; *P*=.98; *ƞ*^2^=0.001).

#### Subjects

To observe how gender and degree of involvement influence individual preferences, this experiment recruited 158 citizens in the city center of Shenzhen, China. The participants were not necessarily actual patients during the experiment. Of the 158 respondents, 80 were men and 78 were women. We recruited only those participants who were older than 18 years. The age distribution was weighted toward adults, with 55.1% (n=87) aged 18-25 years, 22.8% (n=36) aged 26-30 years, and 16.5% (n=26) aged 31-40 years for both men and women. The respondents were assigned to either high-involvement (n=75, 48%) or low-involvement (n=83, 53%) groups. The data showed that the majority of respondents had a bachelor’s degree (n=91, 57.6%), and less than 5% (n=7) had an educational level of junior high school or below. The data also indicated that the largest group of respondents were students (n=58, 36.7%), followed by those working in the commercial or service industry (n=31, 19.6%). Less than 1% (n=1) were retired. Tables 3 and 4 show detailed information on educational level and occupational types.

Respondents were asked to examine the names and photographs of 3 doctors and asked to select 1 from whom they would like to receive treatment. Then, they completed a questionnaire. A 7-point Likert scale was used in this experiment to measure the perceived severity of the disease. We used a 7-point Likert scale, adapted from previous studies on positive emotions caused by physical attractiveness [56,57] and research about the evaluation of attraction and ability [58], to evaluate the physical attractiveness of the physicians. Finally, respondents were asked to provide some relevant demographic information about their age, gender, educational background, and occupation.

**Table 3:** Distribution of respondents by educational level (study 2; n=158).

Educational level	Frequency, n (%)
Junior high or below	7 (4.4)
High school or vocational	22 (13.9)
Associate’s degree	29 (18.4)
Bachelor’s degree	91 (57.6)
Graduate or above	9 (5.7)

**Table 4:** Occupational types of respondents (study 2; n=158).

Occupation type	Frequency, n (%)
Commercial or service industry	31 (19.6)
Industrial or manufacturing	26 (16.5)
Self-employed	10 (6.3)
Retired	1 (0.6)
Students	58 (36.7)
Other	32 (20.3)

### Study 3

#### Overview

All basic conditions and requirements were the same as in study 2. Study 3 examined whether the participants would make a different choice after being provided with extra information. We also sought to further verify that the presence of information about providers’ abilities would reduce the influence of gender on respondents’ preferences.

#### Stimuli

The only difference from the experimental procedure used in study 2 was the adjustment of stimuli. Study 3 added similar ability information about the provider’s professional background and clinical experience below the photograph provided in study 2. The description read: “...graduated from...medical college, ...has participated in many research projects, ...has been working for 5 years, ...is an expert in diagnosis and treatment of common and frequently-occurring diseases...” To minimize confounding factors, the written description of the academic background, clinical experience, scientific research achievements, and areas of expertise of the service providers in the stimuli were very similar.

#### Subjects

Study 3 recruited 150 citizens in the city center of Shenzhen, with equal numbers of respondents by gender (n=75, 50% men and n=75, 50% women). The participants were not necessarily actual patients during the experiment. The age distribution was similar to that of study 2, with a high concentration of young adults: 54% (n=81) aged 18-25 years, 26.7% (n=40) aged 26-30 years, and only 12% (n=18) aged 31-40 years. The high- and low-involvement groups included 74 (49.3%) and 76 (50.7%) respondents, respectively. The data indicated that among the 150 respondents, the majority had a bachelor’s degree (n=82, 54.7%). This was followed by those who had completed high school, vocational school, or technical school (n=26, 17.3%), and then by those with an associate degree (n=25, 16.7%). The data also indicated that among the 150 respondents, the largest group was students, comprising 44% (n=66) of the sample. This was followed by those working in the commercial or service industry, who made up 26.7% (n=40) of the respondents. Those in industrial or manufacturing roles accounted for 7.3% (n=11), and self-employed individuals made up 4.7% (n=7). Tables 5 and 6 show detailed information on educational level and occupational types.

**Table 5:** Distribution of respondents by educational level (study 3; n=150).

Educational level	Frequency, n (%)
Junior high or below	11 (7.3)
High school or vocational	26 (17.3)
Associate’s degree	25 (16.7)
Bachelor’s degree	82 (54.7)
Graduate or above	6 (4)

**Table 6:** Occupational types of respondents (study 3; n=150).

Occupation type	Frequency, n (%)
Commercial or service industry	40 (26.7)
Industrial or manufacturing	11 (7.3)
Self-employed	7 (4.7)
Retired	4 (2.7)
Unemployed	1 (0.7)
Students	66 (44)
Other	21 (14)

## Results

### Study 1

The individual item scores within a single scale were computed to yield an average score, serving as a composite measure for that particular scale. The results revealed that participants perceived doctors in the high attractiveness condition to be more attractive than those in the moderate attractiveness condition (mean_high_ 5.62, SD 1.03 vs mean_moderate_ 4.72, SD 1.36; *F*_1,280_=39.1; *P*<.001; *ƞ*^2^=0.12). This suggested that our manipulation was successful.

We conducted an ANOVA to evaluate our hypotheses. The results showed that the physical attractiveness did not have a significant main effect on perceived competence (mean_high_ 5.28, SD 0.98 vs mean_moderate_ 5.10, SD 1.12; *F*_1,278_=2.32; *P*=.13; *ƞ*^2^=0.008). In consistence with our expectation, we observed a significant interaction effect of physical attractiveness and gender match on perceived competence (*F*_1,278_=5.95; *P*=.015; *ƞ*^2^=0.021). In particular, when the doctor and the participant were of different genders, attractive doctors were perceived to be more competent in the job than less attractive doctors (mean_high_ 5.45, SD 0.93 vs mean_moderate_ 4.95, SD 1.10; *F*_1,133_=8.08; *P*=.005; *ƞ*^2^=0.057). However, when the doctor and the participant were of the same gender, attractiveness did not produce any positive effect on perceived competence (mean_high_ 5.09, SD 1.01 vs mean_moderate_ 5.21, SD 1.13; *F*_1,145_=0.41; *P*=.52; *ƞ*^2^=0.003; see Tables 7 and 8 for detailed information).

Physical attractiveness had a positive effect on intention to select the doctor (mean_high_ 5.49, SD 1.13 vs mean_moderate_ 5.10, SD 1.32; *F*_1,278_=5.10; *P*=.025; *ƞ*^2^=0.018). There was also a significant interaction effect of physical attractiveness and gender match on intention to select the doctor (*F*_1,278_=3.96; *P*=.048; *ƞ*^2^=0.014). More specifically, participants were more likely to select attractive doctors than less attractive doctors of the opposite gender (mean_high_ 5.49, SD 1.17 vs mean_moderate_ 4.86, SD 1.36; *F*_1,133_=8.15; *P*=.005; *ƞ*^2^=0.058). In contrast, when the participants and the doctor were of the same gender, there was no difference in intention to select between more attractive and less attractive doctors (mean_high_ 5.31, SD 1.07 vs mean_moderate_ 5.27, SD 1.27; *F*_1,145_=0.40; *P*=.84; *ƞ*^2^<0.001).

Next, we performed a moderated mediation analysis using Hayes PROCESS (model 7) with 10,000 bootstrap samples. To rule out alternative explanations, we also examined the mediating effect of perceived seniority, perceived embarrassment, perceived friendliness, and perceived willingness to help. The results showed that the moderation effect of gender match on physical attractiveness on intention to select was mediated only by perceived competence (point estimation=0.34, 95% CI 0.0622-0.6593) but not by perceived seniority (point estimation=0.09, 95% CI –0.0601 to 0.2763), anticipated embarrassment (point estimation=0.01, 95% CI –0.0196 to 0.0663), perceived friendliness (point estimation=–0.01, 95% CI –0.0912 to 0.0477), and perceived willingness to help (point estimation=–0.001, 95% CI –0.1395 to 0.1423). In particular, when participants and doctors were of different genders, physical attractiveness led to higher perceived competence of the doctor, thereby increasing the intention to select the doctor (point estimation=0.27, 95% CI 0.0776-0.4994). However, when there was no difference in gender, such an indirect effect was insignificant (point estimation=–0.06, 95% CI –0.2716 to 0.1233).

We conducted a supplementary analysis in which we controlled for age. The results of this age-adjusted analysis remained consistent with our initial findings. Specifically, after accounting for age differences, the interaction effect of gender match and physical attractiveness on perceived competence (*F*_1,277_=4.81; *P*=.03; *ƞ*^2^=0.017) and user intention (*F*_1,277_=2.32; *P*=.006; *ƞ*^2^=0.027) remained stable. Also, the moderated mediation effect was significant (point estimation=0.55, 95% CI 0.1598-0.9762). Therefore, hypothesis 1 was supported.

**Table 7:** Descriptive statistics for perceived competence scores based on physical attractiveness and gender match.

Physical attractiveness and gender match		Score, mean (SD)	Participants, n
**Moderate**			
	Concordance	5.21 (1.13)	82
	Discordance	4.95 (1.10)	59
	Total	5.10 (1.12)	141
**High**			
	Concordance	5.09 (1.01)	65
	Discordance	5.45 (0.93)	76
	Total	5.28 (0.98)	141
**Total**			
	Concordance	5.16 (1.08)	147
	Discordance	5.23 (1.04)	135
	Total	5.19 (1.06)	282

**Table 8:** Descriptive statistics for intention scores based on physical attractiveness and gender match.

Physical attractiveness and gender match			Score, mean (SD)		Participants, n
**Moderate**					
	Concordance	5.27 (1.26)		82	
	Discordance	4.86 (1.35)		59	
	Total	5.10 (1.31)		141	
**High**					
	Concordance	5.31 (1.07)		65	
	Discordance	5.49 (1.17)		76	
	Total	5.40 (1.12)		141	
**Total**					
	Concordance	5.29 (1.18)		147	
	Discordance	5.21 (1.28)		135	
	Total	5.25 (1.23)		282	

### Study 2

A manipulation check on the perceived severity of the disease showed that the scores of the low-involvement group (mean 3, SD 1.47) were significantly lower than those of the high-involvement group (mean 4.45, SD 1.60; t_158_=–5.95; *P<*.001; *ƞ*^2^=0.185). This indicates that the different degree (high vs low) of involvement was successfully manipulated. A 1-way ANOVA was used for a manipulation check on physical attractiveness. The results showed that physical attractiveness of doctors was successfully manipulated (mean_high PA_ 5.61, SD 1.13; mean_middle PA_ 4.77, SD 0.94; mean_low PA_ 3.88, SD 1.28; *F*_1,58_=98.24; *P*<.001; *ƞ*^2^=0.499).

A chi-square test was used to examine hypothesis 1. The result showed that men and women had a significant difference in selecting the doctors with 3 different physical attractiveness levels (^2^_2_=14.165; *P*<.001; =0.299). Furthermore, this research tried to identify the level of physical attractiveness upon which this kind of difference exists. Therefore, a post hoc test was conducted. The analysis finds that adjusted standardized residual of gender preference has a significant difference on high-level (N_men choose high physical attractiveness_=48, N_women choose high physical attractiveness_=29; *z* score_abs_=2.9, *z* score_abs_>1.96; *P*=.004) and middle-level (N_men choose middle physical attractiveness_=25, N_women choose middle physical attractiveness_=47; *z* score_abs_=3.7, *z* score_abs_>1.96; *P*<.001) service providers, while gender has an insignificant effect on those with low physical attractiveness (N_men choose middle physical attractiveness_=7, N_women choose middle physical attractiveness_=2; *z* score_abs_=1.7, *z* score_abs_<1.96; *P*=.09). This indicated that in terms of different physical attractiveness in service providers, men (vs women) preferred highly attractive women service providers, whereas women (vs men) preferred moderately attractive providers; however, there was no difference between men and women’s choice for the low-attractiveness provider. Thus, hypothesis 1 is supported.

To further investigate how involvement in a credence service scenario influences men’s (vs women’s) preferences for different physical attractiveness in service providers, a post hoc chi-square test and an analysis of logistic regression were used. The test found that involvement moderated the effect of gender on preferences (β_gender×involvement_=–18.39; Wald=662.36; *P*=.003). The results illustrated that in the low-involvement condition, the effect of gender on preference was reduced. In particular, the effect of gender on individual preferences existed only in the high-involvement scenario (^2^_2_=9.78; *P*=.008). For the low-involvement scenario, there was no gender effect (^2^_2_=4.82; *P*=.09). Therefore, hypothesis 3 was supported. In the high-involvement condition, compared with women, men preferred the highly attractive service provider (N_men choose high physical attractiveness_=19, N_women choose high physical attractiveness_=12; *z* score_abs_=2.1, *z* score_abs_>1.96; *P*=.04); compared with men, women were more likely to select a service provider with moderate physical attractiveness (N_men choose middle physical attractiveness_=13, N_women choose middle physical attractiveness_=28; *z* score_abs_=2.9; *z* score_abs_>1.96; *P*=.004; Table 9).

**Table 9:** Influence of gender on consumers’ preferences for service providers with different levels of physical attractiveness.

Involvement	Gender	High physical attractiveness	Middle physical attractiveness	Low physical attractiveness
High involvement	Men	19	13	3
High involvement	Women	12	28	0
Low involvement	Men	29	12	4
Low involvement	Women	17	19	2

### Study 3

A manipulation check on the perceived severity of the disease showed that the scores of the low-severity group (mean 5.17, SD 1.14) were significantly lower than those of the high-severity group (mean 3.24, SD 1.13; *t*_148_=–10.40; *P*<.001; *ƞ*^2^=0.422). This indicated that the degree (high vs low) of severity was successfully manipulated. A 1-way ANOVA was used for a manipulation check on physical attractiveness. The data showed that there was a significant difference between the 3 levels of physical attractiveness (mean_high PA_ 5.81, SD 0.90; mean_middle PA_=4.73, SD=0.94; mean_low PA_ 3.82, SD 1.18; *F*_1,50_=144.50; *P*<.001; *ƞ*^2^=0.661). Thus, the physical attractiveness of the 3 doctors was successfully manipulated.

We used a post hoc chi-square test and a logistic regression model to test our hypothesis. The chi-square test results showed that there was no significant difference in the physical attractiveness preferences of men and women (χ^2^_2_=1.147; *P*=.56; *P*>.05; =0.087) when they were provided with extra information about the service provider’s qualification. A further post hoc chi-square test showed that in both the high-involvement (χ^2^_2_=1.730; *P*=.421; *P*>.05; =0.151) and low-involvement (χ^2^_2_=0.046; *P*=.98; *P*>.05; =0.025) scenarios, the effect of gender on preferences disappeared after respondents were given information on the service provider’s abilities; Table 10).

**Table 10:** Influence of gender on consumers’ preferences for service providers (including ability information).

Involvement	Gender	High physical attractiveness	Middle physical attractiveness	Low physical attractiveness
High involvement	Men	10	22	7
High involvement	Women	12	22	3
Low involvement	Men	19	13	4
Low involvement	Women	21	13	4

## Discussion

Study 1 provided evidence to support hypothesis 1. In the context of digital doctor consultation, gender influences people’s preferences for attractive health care providers. People are more likely to select a more (vs less) attractive doctor of the opposite (vs same) gender. This can be explained by the perceived competence of the doctor. In study 1, we adopted a separate evaluation method. On one hand, this method helps to rule out confounding factors and provides strong support for the hypothesis. However, in reality, when patients search for a digital doctor consultation, they may see a list of all available doctors and select the one they prefer. Therefore, in study 2, we adopted a joint evaluation method. Furthermore, we also manipulated the level of disease severity. The results of the second study show that when a service provider’s profile picture was provided to consumers, the consumers’ preferences were greatly influenced by the provider’s gender. The impact of this gender stereotype existed only in the high-involvement condition. If involvement was low, consumers did not have higher requirements for the abilities of medical service providers and thus relied less on their physical attractiveness to infer the ability of the service provider. The results of study 3 illustrated that when extra qualification information was provided, the influence of gender on individual preferences for the service provider’s attractiveness disappeared in both the high- and low-involvement conditions. It is important to note that the context of the research was based in China, a leading country in providing digital consultation services. According to data from the China Internet Network Information Center, by June 2023, the number of users accessing digital medical services in China had surged to 364 million, a rise of 1.62 million since December 2022. The data further reveal that the number of digital hospitals in China has surpassed 3000. These hospitals have offered digital diagnostic and treatment services to more than 25.9 million patients [59]. Thus, the participants in this study were familiar with digital doctor consultations. With the COVID-19 pandemic, an increasing number of European countries have started developing digital consultation services in health care. For instance, Estonia has already begun offering digital services to patients in remote areas. Therefore, the findings from this study can offer significant insights to other countries.

This research contributes to the literature on medical information systems research. First, this study further enhances our understanding of patients’ behavior in digital doctor consultations. As a result of the information asymmetry, patients are always looking for other signals to aid in their decision-making [18]. The lack of trust is one of the biggest barriers to digital services [60]. Besides professional status and service feedback, which are known as important signals for patients’ doctor selection [47], this study has shown that physical attractiveness is also a crucial signal for decision-making in the digital doctor consultation context. This study also helps to deepen the understanding of SAB in the context of digital health care services. Previous studies on SAB focus on contexts such as job interviews [61]. More recently, the literature has increasingly focused on general services, such as education services [15]. In addition, this study sheds light on the boundary condition of SAB. Our findings indicate that the degree of disease severity influences the effect of gender on preferences for attractive service providers. Based on the elaboration likelihood model, consumers with low disease involvement tend to rely on peripheral cues to make choices, which means that they may automatically choose a more attractive service provider. Nevertheless, our research points out that this assumption only holds for both men and women when qualification information is present. When qualification information is absent and information asymmetry exists, men and women react differently to attractive service providers when they are highly involved.

These research findings have some crucial implications for digital health care service management. Providing qualification information of service providers on digital health consultation platforms can mitigate the effect of gender on the individual preference for the attractiveness of a service provider, regardless of the scenario’s level of involvement. In the health care service sector, providing sufficient information to eliminate bias is a useful strategy. In the context of information asymmetry, patients may be influenced by the doctor’s physical attractiveness and make irrational decisions. When marketing telehealth services, hospitals should focus on highlighting doctor expertise, patient satisfaction, and health outcomes instead of relying on doctor attractiveness or demographic characteristics to promote their services. Furthermore, hospitals should develop patient education materials that explain the importance of choosing a doctor based on their qualifications and expertise, rather than their appearance. Also, digital health care platforms could consider implementing features that allow patients to filter and search for doctors based on their qualifications, expertise, and patient satisfaction ratings. This can help patients to make more rational decisions.

This study has some clear limitations, which point, in turn, to avenues for future research. One notable limitation of this study is the restricted age range of both the health care providers depicted in the images and the study participants, who were predominantly younger than 25 years. This lack of age diversity could potentially limit the generalizability of the findings, as perceptions of physical attractiveness and professionalism may vary across different age groups. Although supplementary analyses controlling for age did not significantly alter the results, it remains an open question whether these findings would hold in a more age-diverse sample. Future research should aim to include a broader age range of both health care providers and participants to explore the potential moderating effect of age on the relationships examined in this study.

Another limitation of this study pertains to the clinical scenario presented, which focuses on an acute condition that could potentially be resolved in a single consultation. This may not fully capture the complexities involved when patients are seeking long-term care for chronic conditions. In such cases, the level of disease involvement is likely to be higher, and patients may seek more comprehensive information to assess a doctor’s competence. The dynamics of how gender match and physical attractiveness influence patient choices could thus differ in a chronic care setting. Future research could benefit from exploring these nuances to provide a more holistic understanding.

Another limitation of this study is its focus on China. To enhance the generalizability of our research and generate a more worldwide impact, replication in various countries and across different cultures is necessary. Future research should, for instance, involve recruiting subjects from diverse nations. This is because health care systems vary significantly among countries. Systematic differences in health care structures, policies, and practices can substantially influence how patients evaluate doctors digitally. Consequently, these contextual differences should be carefully controlled for and considered in future studies to ensure the validity and applicability of research findings across different health care systems and cultural backgrounds. Furthermore, the cultural orientation of the patient can also play a role in this situation. Future studies should investigate how varying cultural dimensions, such as collectivism, power distance, and uncertainty avoidance, influence patient preferences and decision-making processes in digital health care settings. For instance, individuals from countries with high uncertainty avoidance may require more concrete information in the doctor’s description to feel comfortable with their choice.

Another important research direction involves examining the impact of other forms of information asymmetry in digital health care settings. For instance, the availability and quality of digital reviews can play a significant role in shaping patient choices. By analyzing the content and credibility of web-based reviews, researchers can better understand how they contribute to patients’ decision-making processes and their perceptions of service providers. In addition, assessing the role of digital reputation management and its influence on patient choices can offer valuable insights into digital health care.

## Abbreviations

SAB: sexual attribution bias
